# RURAL LIVING AND DISABILITY IN OLDER ADULTS: THE ROLE OF ALTERNATIVE SUPPORT RESOURCES AS MEDIATORS

**DOI:** 10.1093/geroni/igac059.1864

**Published:** 2022-12-20

**Authors:** Bernard Steinman, Daniel Barbakoff

**Affiliations:** University of Wyoming, Laramie, Wyoming, United States; Weill Cornell Medical College, New York, New York, United States

## Abstract

Rural communities are often characterized by sparse service environments offering limited care, services, and conveniences that help with daily activities. In lieu of community services that target older adults to assist with aging-in-place, alternative supportive features, including environmental modification and informal social networks may be especially important in rural settings to preserve functional independence. The purpose of this study was to assess the role of alternative support resources as potential mediators between service environments and Activities of Daily Living (ADL) functioning of older adults living in rural settings. Data from the National Health and Aging Trends Study (NHATS) were analyzed. Guided by the International Classification of Functioning, Disability and Health, regression models included covariates for sociodemographics, chronic conditions, mobility functioning, and participation. Service environments were quantified using a measure of the number of services (e.g., help with bathing) available in communities. Two potentially important support features were tested as mediators. Environmental modification was operationalized using indicators of whether homes had been modified (e.g., with features such as grab bars). Size and quality of individuals’ social networks were calculated using indicators of whom participants spoke to about important things in their life. Measures of ADLs served as key dependent variables. Results suggest a negative statistical relationship between service environments and disability that is explained in part by the availability of alternative support resources. Implications are that older adults who live in rural communities may often benefit by employing home modifications and relying on informal care options to meet their needs.

